# Epidemiological pattern of deaths caused by traffic accidents in East Azerbaijan, Iran

**Published:** 2019-08

**Authors:** Ali Jafari-Khounigh, Ilnaz Iranzad Khiyabani, Mohsen Nouri, Jila Mahmoodi

**Affiliations:** ^*a*^Department of Biostatistics and Epidemiology, School of Health, Kerman University of Medical Sciences, Kerman, Iran.; ^*b*^East Azerbaijan Province Health Center, Tabriz University of Medical Sciences, Tabriz, Iran.; ^*c*^Department of Disaster Public Health, School of Public Health, Tehran University of Medical Sciences, Tehran, Iran.; ^*d*^Tabriz Health Center, Tabriz University of Medical Sciences, Tabriz, Iran.

## Abstract

**Background:**

Traffic accidents are one of the main causes of mortality in the world. They are currently estimated to be the ninth leading cause of death across all age-groups globally and are predicted to become the seventh leading cause of death by 2030. Road traffic crashes are a leading cause of death among young people, and the main cause among those aged 15–29 years worldwide. The number of road traffic deaths continues to rise steadily, reaching 1.35 million in 2016, from 1.15 million in 2000. The purpose of the present study is to investigate epidemiologic pattern, and trend of deaths due to accidents in East Azerbaijan province, Iran, from 2012 to 2016, in order to identify its effective determinants to conduct prevention programs.

**Methods:**

This cross-sectional study was conducted using mortality data of province health center from 2012 to 2016. The data of deaths due to traffic accidents were analyzed using descriptive statistics by means of Microsoft Excel 2013 and SPSS 16.

**Results:**

A number of 4529 deaths due to traffic accidents occurred during five years of the study period, which were 4.95% of total deaths of all causes, and the third leading cause of mortality. Of all accident deaths, 79.5% were male and 69.7% took place in urban residents. Most accidents occurred in the summer (30.1%), and in August (10.9%). The highest (20.8%) and the lowest (18.2%) deaths occurred in 2016 and 2014, respectively. However, the highest incidence was in 2012 with 26.28 per 100000, and the lowest incidence in 2014 with 23.15 per 100000. The mean age and SD for males and females were 41.15±20.27 and 41.45±23.73 respectively, and the highest percentage of deaths happened in the age-group 20-29 (18.4%), followed by age-group 30-39 (18.3%). One hundred and seventy-eight (3.9%) accident victims were children under five years of age, one of the most important causes of death for this age group.

**Conclusions:**

Regarding the high incidence of mortality due to accidents, identification and correction of high-risk areas, and strict enforcement of traffic rules are necessary in order to prevent accidents and related deaths.

**Keywords:**

Epidemiology, Death, Traffic Accidents, East Azerbaijan

